# The biomarkers of hyperprogressive disease in PD-1/PD-L1 blockage therapy

**DOI:** 10.1186/s12943-020-01200-x

**Published:** 2020-05-02

**Authors:** Xueping Wang, Fang Wang, Mengjun Zhong, Yosef Yarden, Liwu Fu

**Affiliations:** 1 State Key Laboratory of Oncology in South China, Collaborative Innovation Center for Cancer Medicine, Guangdong Esophageal Cancer Institute, Sun Yat-sen University Cancer Center, Guangzhou, China; 2 Department of Biological Regulation, Weizmann Institute of Science, 76100 Rehovot, Israel

**Keywords:** Immunotherapy, PD-1/PD-L1, Hyperprogressive disease, Biomarker

## Abstract

Immune checkpoint inhibitors (ICIs), such as PD-1/PD-L1 antibodies (Abs) and anti-cytotoxic T lymphocyte antigen 4 (CTLA-4) Abs, are effective for patients with various cancers. However, low response rates to ICI monotherapies and even hyperprogressive disease (HPD) have limited the clinical application of ICIs. HPD is a novel pattern of progression, with an unexpected and fast progression in tumor volume and rate, poor survival of patients and early fatality. Considering the limitations of ICI due to HPD incidence, valid biomarkers are urgently needed to predict the occurrence of HPD and the efficacy of ICI. Here, we reviewed and summarized the known biomarkers of HPD, including tumor cell biomarkers, tumor microenvironment biomarkers, laboratory biomarkers and clinical indicators, which provide a potential effective approach for selecting patients sensitive to ICI cancer treatments.

## Background

In recent years, immunotherapy has been introduced as a breakthrough in cancer treatment. Immune checkpoint inhibitor (ICI) therapies, including anti-PD1, anti-PD-L1, and anti-CTLA-4 therapies, have played a role in enhancing the activities of effective T cells and inhibiting the immunosuppression in the tumor microenvironment [1, 2]. ICI therapies have revolutionized the systemic treatments for advanced tumors, including melanoma [3], non-small cell lung cancer (NSCLC) [4], kidney cancer [5] and squamous cell carcinoma of the head and neck (HNSCC) [6]. Patients accepting ICI therapy have a better survival time and an unprecedented higher cure rate [4] than those treated with conventional therapies.

**Fig. 1:**
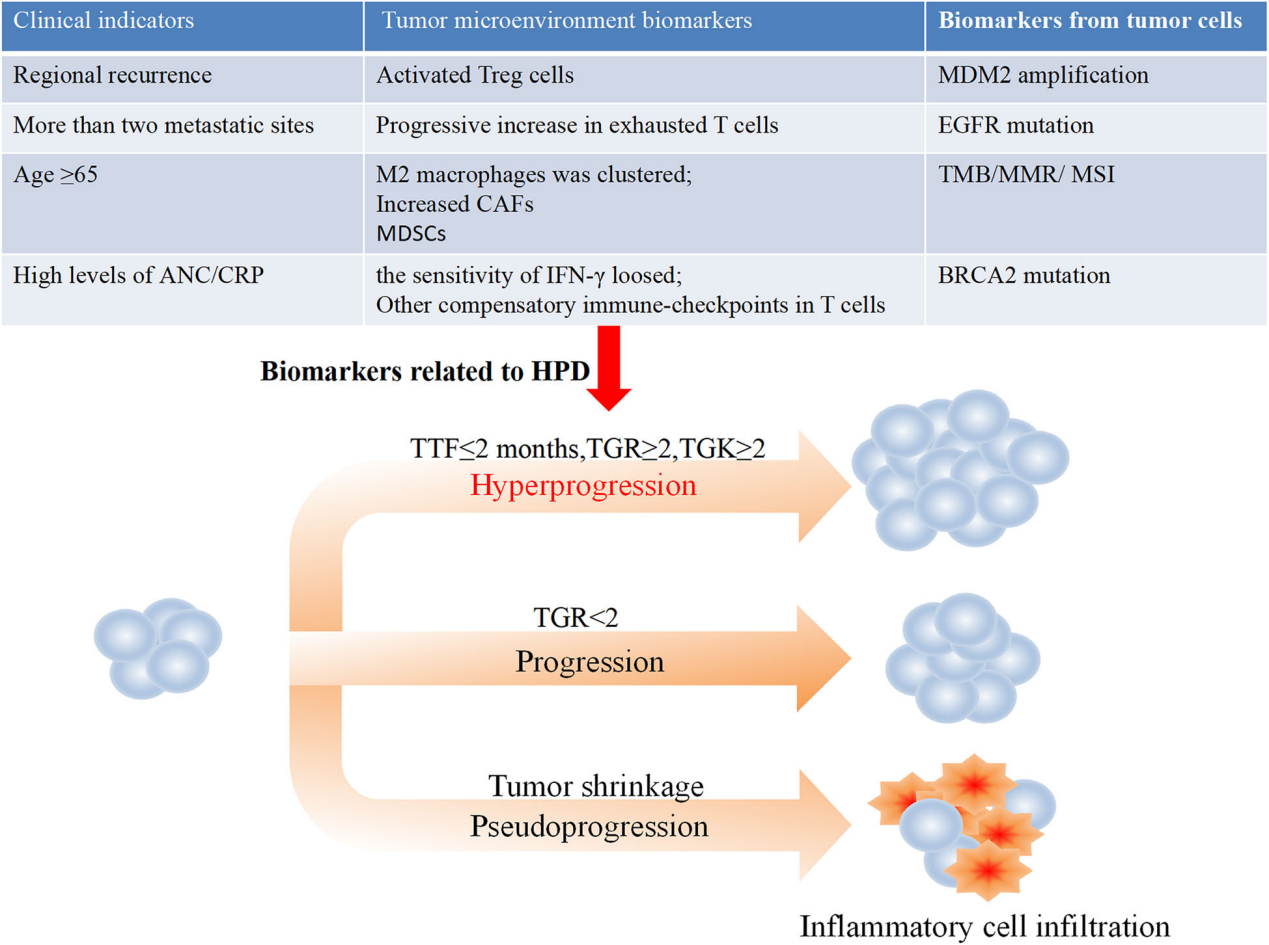
Definition of hyperprogressive disease (HPD): TTF ≤ 2 months, TRG ≥ 2 and/or TGK ≥ 2 (TTF: time to treatment failure; TGR: tumor growth rate; TGK: tumor growth kinetics). The potential biomarkers for HPD after immune checkpoint blockade, including tumor microenvironment biomarkers, clinical indicators and tumor cell biomarkers

Successful clinical trials have enabled wide application of ICIs in the clinic. To date, there are five FDA-approved ICIs for PD-1/PD-L1, including pembrolizumab and nivolumab (anti-PD-1) and atezolizumab, durvalumab and avelumab (anti-PD-L1). Moreover, many studies have shown that the reactivities of patients given ICIs or classical chemotherapy differ significantly. First, a long duration in tumor shrinkage (partial/complete response: PR/CR) occurred in 10–30% of patients on ICI therapy (anti-PD-1/PD- L1 and/or anti-CLTA-4), which is much higher than that presented with other therapeutic regimens, and the overall survival (OS) was unprecedentedly improved [7]. Second, some patients underwent pseudoprogression, assessed by imaging; the tumor burden showed a primary surge followed by shrinkage (usually at 4 weeks confirmed), which also resulted in a survival benefit. With the appearance of pseudoprogression, the development of irRC [8], irRECIST [9] and iRECIST [10] was motivated, which have been used to estimate the response to ICI treatment. Finally, progression of disease, especially hyperprogression, is a novel pattern of progression observed with ICI treatment (Fig. 1).

In ICI treatment, several studies have reported that progression at an accelerated and unexpected rate and volume might present in more than a few patients, which often leads to dramatically reduced survival durations. This condition was then termed hyperprogressive disease (HPD), which has been observed in various types of tumors. Clinical or molecular factors for HPD prediction are urgently needed to evade the potential dangers. However, in ICI therapy, the mechanism and characteristics of HPD remain extremely unclear.

In this review, a clinically oriented and latest overview has been put forward on the basis of various studies on HPD. Meanwhile, the potential mechanisms, predictive biomarkers and diagnostic methods have also been integrated, which provides a foundation for the management of HPD patients.

### Incidence of HPD

As a new type of tumor response, HPD is characterized as an unexpected and fast progression in tumor volume and rate, which is connected with an inferior outcome. The definition is as follows [11]: (1) TTF (time to treatment failure), the time between the beginning of ICI therapy to the interruption with no reason is within 2 months (TTF ≤ 2 months); (2) TGR (tumor growth rate), in contrast with the pretherapy images, the patient tumor volume is doubled or increased (TGR ≥ 2); and/or TGK (tumor growth kinetics), objective lesion changes in unit interval determined by evaluation of the largest diameters according to RECIST (TGK ≥ 2).

HPD was first described in a study by Champiat et al. [12]*.* The retrospective study included patients with various tumors in phase 1 trials, (melanoma, lung cancer, renal cancer, colorectal cancer, and urothelial cancer, among other types) that showed a 9% increase in the incidence of HPD (12/131) during treatment with PD-1/PD-L1 inhibitors compared with the chemotherapeutic group. Then, retrospective data and several clinical cases of HPD were also reported during anti-PD-1/PD-L1 therapy. HPD incidence is not limited to specific tumors in accordance with these respective observations. It was found that 13.8% (56/406) of patients with PD-1/PD-L1 blockade therapy underwent HPD (based on TGR ≥ 2) in advanced NSCLC [13]. Another group retrospectively observed a 7% HPD incidence in 182 patients with ICI treatment in a phase 1 study based on the TGR criterion in multiple cancer types [14]. Saâda-Bouzid et al. [15] found that 29% (10/34) of advanced head and neck squamous cell carcinoma (HNSCC) patients given ICI treatment exhibited HPD according to TGR ≥ 2. A study performed by Lo Russo G et al. [11] declared that 25.7% (39/152) of NSCLC patients treated with an ICI met the HPD norm (TTF ≤ 2 months, TGK ≥ 2). Four percent (6/155) of 155 patients with many types of tumors had HPD, which was defined as tumor growth > 40% and a TTF ≤ 2 months. Matos et al. [16] observed HPD in 15% of 214 (32/214) patients in phase I studies treated with ICI therapy, the standard of which was based on RECIST (tumor volume enlargement > 40% and a TTF ≤ 2 months) (Table 1).

**Table 1:** Relevant HPD studies in patients receiving ICB therapy

HPD biomarker (Incidence)	Number (Percentage)	Histology	HPD definition	Ref
Age ≥65, 7/36 (19%) < 64, 5/95 (5%)	12/131 (9%)	Melanoma (45), lung (13), renal (9), colorectal (8), urothelial (8), others (48)	≥2-fold increase in TGR, RECIST progression	Champiat et al. *2017*
Regional recurrence Yes, 9/10 (90%) No, 1/10 (10%)	10/34 (29%)	Recurrent and/or metastatic head and neck squamous cell carcinoma	TGK ≥ 2.	Saada-Bouzid et al. *2017*
Metastatic sites > 2, 35/56 (62.5%) < 2, 21/56 (37.5%)	56/406 (13.8%)	Advanced NSCLC	≥2-fold increase in TGR	Ferrara et al. 2018
Sex Male, 2/99 (2.0%) Female, 10/83 (12.0%)	12/182 (6.5%)	Head and neck (10), gynecological (9), lung (8), gastrointestinal (8), genitourinary (7), others (13)	≥2-fold increase in TGR, RECIST progression	Kanjanapan et al. 2019
*MDM2* family amplification Yes, 4/6 (67%) *EGFR* aberrations Yes, 2/10 (20%)	6/155 (4%)	Melanoma (51), NSCLC (38), Squamous cell carcinoma of head and neck (11), cutaneous squamous cell carcinoma (9), renal cell carcinoma (6), colorectal cancer (5)	TTF < 2 months, > 50% increase in TMB and > 2-fold increase in progression pace	Kato et al. *2017*
Elevations in ANC level 4490/μl vs. 7740/μl (non-HPD vs. HPD) Elevations in CRP level 0.4 mg/dl vs. 8.3 mg/dl (non-HPD vs. HPD)	13/62 (21%)	AGC	≥2-fold increase in TGR, RECIST progression	Sasaki et al. 2019
PD-1^+^ Treg cells in tumor tissues	4/36 (11.1%)	AGC	TTF < 2 months, ≥2-fold increase in TGR, and > 2-fold increase in progression speed	Kamada et al. 2019
MPO^+^ myeloid cells within the tumor IHC in HPD: 3.5 (0.1–6.0) PD-L1 expression in tumor cells IHC in HPD: 1.0 (0.0–10.0)	39/152 (25.7%)	NSCLC	TTF < 2 months, ≥2-fold increase in TGR, at least two new lesions in an organ, spread to a new organ, ECOG PS ≥ 2, at least three of the above criteria and RECIST 1.1 progression	Lo et al. 2019
% of CCR7^−^CD45RA^−^ in CD8+ T cells Low frequencies %TIGIT^+^ in PD-1^+^CD8^+^ T cells High frequencies	TTF: 98/263 (37.3%), TGR: 54/263(20.5%), TGK: 55/263 (20.9%),	NSCLC	TTF < 2 months, ≥2-fold increase in TGR, TGK ≥ 2, RECIST1.1 progression	Kim et al. 2019

Abbreviations

*TGR* Tumor growth rate*TGK* Tumor growth kinetics*TTF* Time to treatment failure*NSCLC* Non-small cell lung cancer*HNSCC* Squamous cell carcinoma of the head and neck*AGC* Advanced gastric cancer*ANC* Absolute neutrophil count*CRP* C-reactive protein*MDM2/4* Murine double minute 2/4*TMB* Tumor mutational burden*EGFR* Epidermal growth factor receptor

The above findings indicate that patients with HPD allocated to ICI treatment experienced a poor prognosis, such as a faster decline in progression-free survival (PFS) and overall survival (OS) compared with those treated with conventional therapies. However, because of patient heterogeneity, different sample sizes and selection bias, the retrospective literature concerning HPD has limitations. Further prospective studies in various tumors may be needed to provide comprehensive HPD data.

### Biomarkers associated with HPD

According to the above studies, HPD has been found in various cancers, such as NSCLC, HNSCC, melanoma, lymphoma, and colorectal, urothelial, biliary tract and ovarian carcinoma. Furthermore, no association has been found between HPD and other clinical characteristics, including blood composition, the occurrence of corticosteroids at baseline (estimated by RECIST), previous systemic treatment, routinely assessed biochemical parameters (such as lymphocyte count and cellular populations), PD-1/PD-L1 expression, or the Royal Marsden Hospital (RMH) score [17]. Patients who obtained benefits from ICI should be selected, while patients with HPD are ruled out, and the mechanisms of HPD, which are complex, dynamic and interdependent, should be analyzed.

**Fig. 2:**
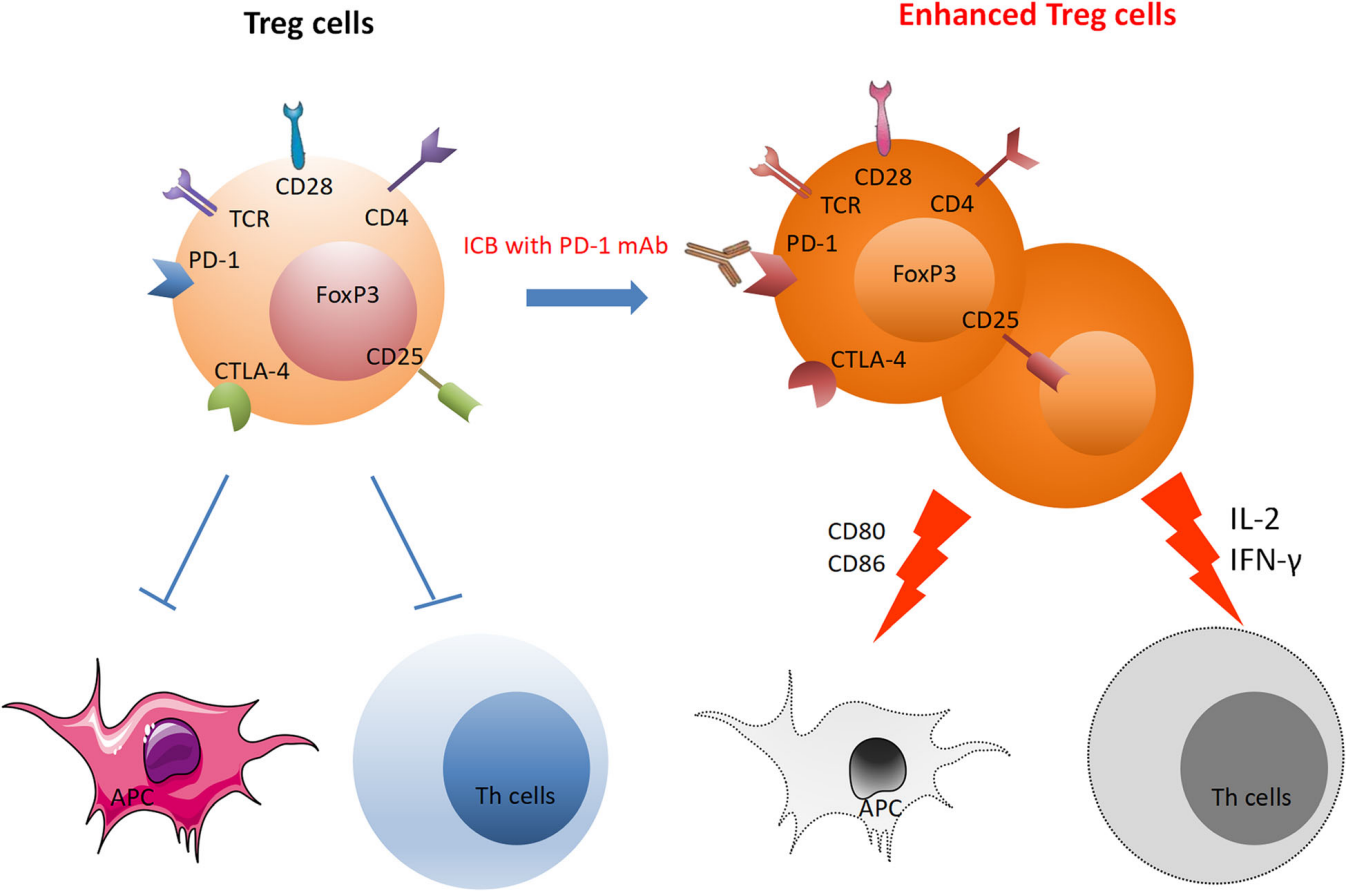
Possible biomarkers in the tumor microenvironment after ICI therapy, including exhausted T cells, Treg cells, M2 TAMs, and MDSCs

To avoid the damage induced by ICI treatment, developing biomarkers for HPD prediction is quite necessary. As shown in Table 2 and Fig. 1, many biomarkers have been discovered to be associated with HPD, including tumor cell biomarkers, tumor microenvironment biomarkers (Fig. 2), laboratory biomarkers, and clinical indicators.

**Table 2:** The possible mechanism of biomarkers in HPD after ICB therapy

Biomarker	Description	Mechanism
**Tumor cell biomarkers**		
MDM2 family	MDM2 is overexpressed by amplification	Hyperexpression of MDM2 might be triggered by amplification during ICB therapy through IFN-γ, especially JAK-STAT signaling that increases IRF-8 expression. Overexpression of MDM2 due to amplification associated with metastasis and formation of the transfer site.
EGFR mutation	EGFR gene mutations and protein overexpression	EGFR mutation is related to upregulated expression of PD-1, PD-L1, CTLA-4 and immunosuppressive cells, such as Treg cells and macrophages; EGFR gene mutations and protein overexpression are associated with cancer growth through activation of downstream pathways: the MAPK pathway, PI3K/AKT pathway and JAK/STAT pathway
BRCA2 mutations	Enrichment for BRCA2 mutations	As the LOF mutation, BRCA2 mutations might impair dsDNA break repair mechanisms and homologous recombination, which might induce specific mutational features related to anti-PD-1 responsiveness. Conversely, it is related to HPD.
MMR/MSI	MMR deficiency leads to accumulation of mutations	More potential neoantigens were produced by the accumulation of mutations of MMR deficiency, which upregulated TIL density, increased TMB, elevated PD-L1 expression, and induced a greater immune response to the tumor. Conversely, it is related to HPD.
TMB	An independent biomarker that is predictive of ICB outcomes	The accumulation of genomic alterations generates neoantigens at the protein level, which may be recognized by the patient’s immune system as nonself or foreign antigens. Neoantigenicity is measured by TMB. Conversely, it is related to HPD.
**Tumor microenvironment biomarkers**		
**Immunological cells**		
Treg cells	Activated Treg cells enhance suppressive activity	Hamper activation of effector T cells, resulting in more Treg cells; inhibit IL-2 release and absorb it; many factors such as adenosine and IDO are upregulated
Exhausted T cells	Progressive increase in exhausted T cells	Upon blocking of PD-1, the compensatory immune-checkpoints (PD-1, TIM3, LAG3 and TIGIT) might overexpressed and regulate local immune suppression and escape
Dendritic cells	Generating the anti-tumor response by T cells	The response of T cells could be inhibited through PD-L1 by DCs; cytokines, such as TGF-β, IL-6 and IDO, inhibit the activity of DCs, thus having a negative regulatory effect on T cells
MDSCs	High frequencies of MDSCs related to ICI resistance	Impair the activity of effector T cells, induce expansion of Treg cells, reduce the functions of NK cells, secrete cytokines (IDO, VEGF, MMP9 et al.)
M2 macrophages	Triggering of clustered M2 TAMs	The response of T cells could be inhibited through PD-L1 by M2 TAMs; the binding between specific immunophenotypes through ICB and FcR might trigger clustering of M2 TAMs, which could induce more aggressive protumorigenic behavior by upregulating functional reprogramming in M2 TAMs
**Nonimmunological cells**		
CAFs	Related to immunosuppression	Recruit monocytes that encompass immunosuppression and enhance the motility of tumor cells; induce differentiation of M1 TAMs into M2 TAMs; inhibit T cell immunity through neutrophils
C**ytokines and inflammatory factors**		
IFN-γ	Loss of sensitivity to IFN-γ	Molecules in the IFN-γ pathway, including IFNGR1/2, JAK1/2, STAT1, PI3K-AKT, and IRF1, were mutated or downregulated, thus decreasing the expression of PD-L1
Other compensatory immune checkpoints in T cells	PD- L2/soluble PD-1	PD-1 blockade can induce inhibition of T cells through the combination of PD-1 and PD-L2; soluble PD-1 fusion protein might inhibit the activity of bone marrow-derived DCs and increase the secretion of IL-10
**Laboratory biomarkers**		
ANC/CRP	ANC and CRP were significantly higher in the HPD group than in the non-HPD group	The upregulation of the ANC might be used to reflect the release of premature myeloid cells from the bone marrow, such as MDSCs, which are related to tumor invasion and metastasis. MDSC counts are also positively correlated with CRP levels
**Clinical indicators**		
Regional recurrence in an irradiated field	RSCCHN patients with regional recurrence had HPD	Radiotherapy might be related to ICB treatment failure by regulating the tumor microenvironment through a decrease in TILs and the main cytokines and an increase in PD-L1 transcripts
More than two metastatic sites	HPD was more frequent in patients who had more than two metastatic sites of advanced NSCLC	More aggressive tumor phenotypes might be related to HPD
Age ≥ 65	More patients of advanced age (≥65) had HPD than younger patients.	Immunosenescence: age-related thymic atrophy connected with lowing the T cell immunity, which plays a key role in autoimmune disease, infection, and tumors

Abbreviations

*MDM2/4* Murine double minute 2/4*EGFR* Epidermal growth factor receptor*BRCA2* Breast cancer susceptibility gene 2*MMR* Mismatch repair*MSI* Microsatellite instability*TMB* Tumor mutational burden*MDSCs* Myeloid-derived suppressor cells*CAFs* Cancer-associated fibroblasts*IFN-γ* Interferon-γ*ICI* Immune checkpoint inhibitors*ANC* Absolute neutrophil count*CRP* C-reactive protein

#### Tumor cell biomarkers

##### Amplification of murine double minute 2/4 (MDM2/4)

MDM2 amplification has been shown to be associated with HPD. In cell lines of spontaneously transformed mice, MDM2 was initially found to be overexpressed based on amplification as an oncogene subset [18] and plays a key role in promoting tumor growth by inhibiting gene transactivation of the tumor suppressor p53. Overexpression of MDM2 is related to an inferior prognosis in various tumors, such as stomach, lung, esophagus and breast cancer; leukemia; glioblastoma; liposarcoma; and other treatment-resistant tumors. MDM2 is also associated with tumor metastasis and formation of the transfer site (such as in prostate, colon and breast cancers and osteosarcomas) [19–21].

To investigate valuable genomic features related to HPD, the genomic profiles of 155 patients were analyzed via next-generation sequencing (NGS), and Kato et al. [22] reported that worse outcomes were associated with alterations in MDM2/4 (*p* = 0.02), DNMT3A (*p* = 0.04) and EGFR (*p* = 0.02); especially, 67% (4/6) of patients with MDM2/4 amplifications underwent HPD. The possible mechanism is that ICI treatment triggers hyperexpression of MDM2 amplification by promoting the expression of IRF-8 (interferon regulatory factor 8) and binding to the MDM2 promoter through JAK-STAT signaling. Therefore, MDM2 inhibitors could be a potential therapy to control the development of HPD. Meanwhile, amplification of MDM2/4 might be a useful marker of HPD.

##### EGFR mutation

As a member of the HER (human epidermal receptor) family, EGFR (epidermal growth factor receptor) is a transmembrane tyrosine kinase receptor (RTK). EGFR has roles in cell growth, proliferation and differentiation through many cell signaling pathways, including the PI3K (phosphatidylinositol-3-kinase)/AKT (protein kinase B) pathway, RAS (rat sarcoma)/RAF (rapidly accelerated fibrosarcoma)/MAPK (mitogen-activated protein kinase) pathway, and JAK (janus kinase)/STAT (signal transducers and activators of transcription) pathway. EGFR gene mutations and EGFR protein overexpression are associated with tumor growth through activation of downstream pathways, especially in lung cancer [23]. HPD has been found in 20% (2/10) of patients with EGFR alterations and has led to worse outcomes (Table 1). Gainor et al. observed that EGFR mutations and ALK rearrangement were related to low response rates to ICI therapy in lung cancer patients [24]. A possible explanation is that EGFR mutations upregulate cell-surface inhibitory receptors (for example, PD-1/PD-L1 and CTLA-4), cytokines (such as IL-6, IL-10 and TGF-β), and immunosuppressive cells (regulatory T cells and macrophages), which can drive innate immune resistance [25]. However, the role of mutant EGFR in HPD requires further investigation.

##### Enrichment for BRCA2 mutations

Hugo et al. [26] reported that responding melanoma patients have more BRCA2 mutations while nonresponding patients have fewer mutations (*P* = 0.002) upon ICI treatment, but the mechanisms are still puzzling. There are six main loss-of-function (LOF) mutation protein domains in BRCA2 that can possibly explain the phenomenon: one in the domain for POLH interaction; one in the region for NPM1 interaction at the N-terminal; and four in the helical domain for FANCD2 interaction. It is interesting that the somatic mutation load of BRCA2 in normal tissue was significantly lower than that in the tumors both in this study and the other two groups of melanoma patients. In general, LOF mutations in BRCA2 might be related to the responsiveness to ICI treatment by affecting the repair of double-strand DNA breaks and homologous recombination, which could result in many unknown effects and specific mutational features.

#### Deficiency of mismatch repair and microsatellite instability

The function of the MMR (mismatch repair) system includes modification insertion, mismatch, and deficiency deletion in base pairing during DNA replication, which has high concordance with microsatellite instability (MSI). Loss of DNA MMR, including MLH1 (MutL homolog 1), MSH2 (MutS protein homolog 2), MSH6 (MutS homolog 6) and PMS2 (PMS1 homolog 2), could be involved in oncogenesis and reduced genomic stability. Le et al. [27] showed that MMR deficiency is related to the responsiveness to ICI treatment in colorectal cancers. Furthermore, they validated this conclusion in 12 different tumor types. One main mechanism is that the potential mutant neoantigen in tumors could result in the clones of T cell expanding rapidly in vivo, which is caused by the mutation accumulation of MMR deficiency. Other possible mechanisms include upregulation of PD-L1 expression, an increase in the TMB, elevated TIL density and more potential immune responses to the tumor [28, 29].

#### Tumor mutational burden (TMB)

Except for some specific genetic alterations, the vast majority of alterations in tumor DNA are considered to be related to responses to ICI therapy. TMB has been used as a surrogate measurement of somatic mutation accumulation. Many clinical studies have reinforced the notion of TMB as an independent biomarker that is predictive of ICI outcomes. Patients with a lower TMB experienced worse response rates and OS than those with a higher TMB. In a prospective randomized clinical trial (Checkmate 026), it was found that PFS was longer in advanced NSCLC patients with a higher TMB [30]. Moreover, a subset of respective studies validated the predictive value of TMB in patients given various ICI therapies and with different cancer types [31]. It is worth noting that TMB cutoffs have been defined differently across studies, testing platforms, and various patient populations. In addition, it is important to acknowledge that cutoffs might differ between tumor types and ICI agents (e.g., > 16 mutations/Mb for atezolizumab in urothelial carcinoma; > 23.1 mutations/Mb for pembrolizumab in NSCLC; and ≥ 13.5, ≥15.8, or ≥ 17.1 mutations/Mb for atezolizumab in NSCLC) [32–34]. A possible explanation is that the accumulation of genomic alterations will generate neoantigens at the protein level, which could be recognized as foreign or nonself antigens by the patient immune system. Neoantigenicity, measured by TMB, is believed to be responsible for the augmentation of the immune response and has an impact on the responses to checkpoint blockade therapy [35–38]. As a valuable biomarker of ICI treatment for tumors, TMB still needs to be further explored more to improve clinic outcomes of patients. The standardization of TMB detection and clinical studies of its use in varying disease states would push forward the approval of TMB as a companion diagnostic for ICI [39]. Thus far, no study has reported the relationship between TMB and HPD.

### Tumor microenvironment biomarkers

#### Immunological cells

##### Regulatory T (Treg) cells

Treg cells are a subset of CD4^+^ cells that specifically express the forkhead box protein P3 (FoxP3) and play a key role in modulating the antitumor T cell responses in the tumor microenvironment. There is accumulating evidence that PD-1 and/or PD-L1 are expressed on Treg cells, and thus, the activity of Treg cells could be influenced by PD-1/PD-L1 blockade. Kamada et al. reported that the activation of Treg cells was enhanced by blockage or deficiency of PD-1 in both humans and mice [40].

**Fig. 3:**
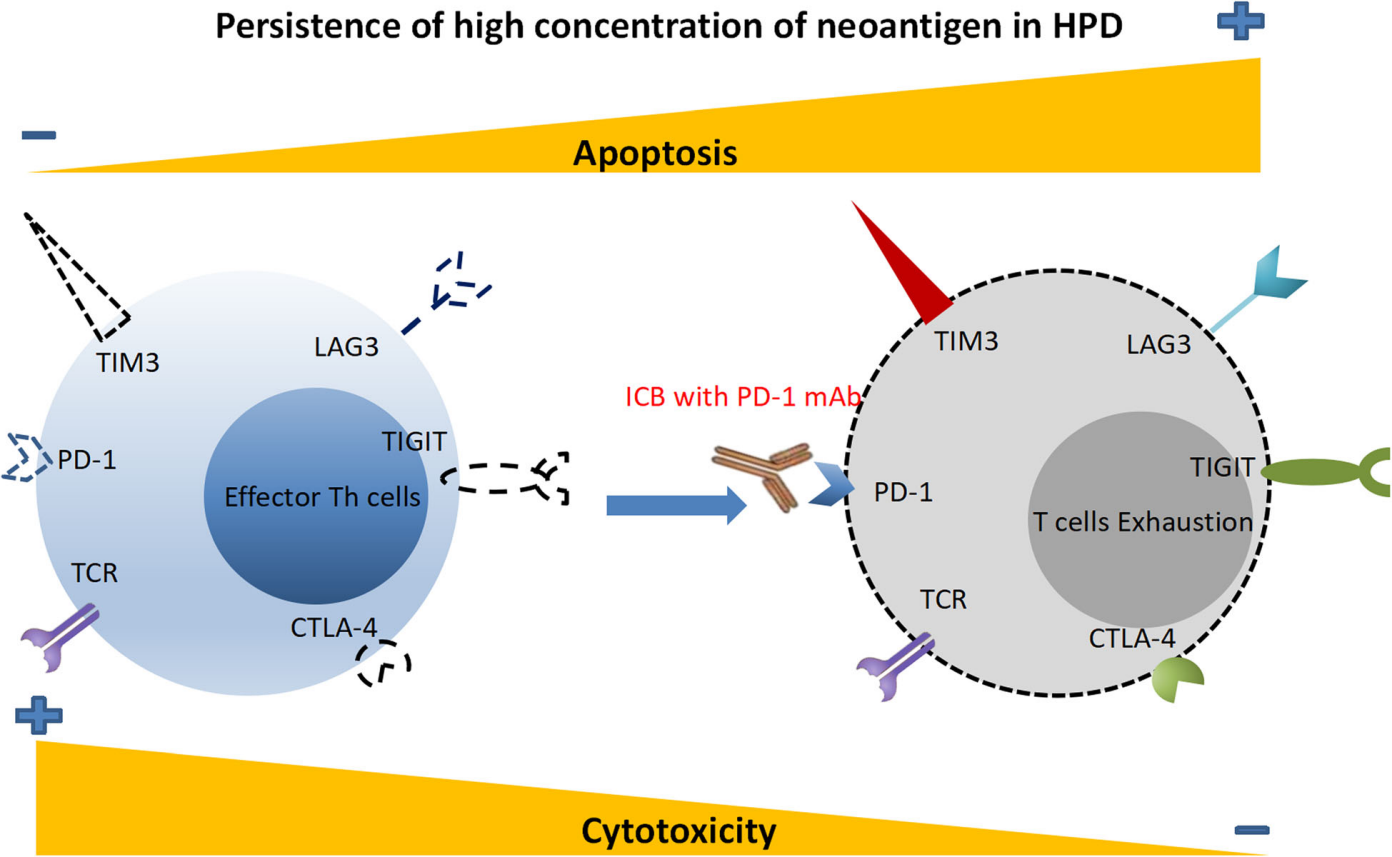
PD-1 blockade significantly enhanced the proliferation and suppression activity of Treg cells: 1. Activated TCR and CD28 signaling enhances Treg cell proliferation and suppression activity. 2. High CTLA-4 expression may recruit more Treg cells. 3. Inhibition of IL-2 release from tumor-reactive T-eff cells and rapid absorption through CD25. 4. Adenosine and indoleamine 2,3-dioxygenase (IDO) are upregulated. 5. IFN-γ is suppressed by Treg cells

The possible mechanisms are as follows: First, PD-1 blockage treatment increases TCR and CD28 signaling in Treg cells, thereby enhancing their proliferation and suppression activity. Strong immune suppression caused by expanded and activated Treg cells hampers the functions of effector T cells, including CD4^+^ T and CD8^+^ T cells [41, 42]. Second, more Treg cells would be recruited by the activated PD-1^+^ Treg cells with high expression of CTLA-4 through restriction of APC activation and access for CD8^+^T cells (downregulation of the costimulatory molecules CD80/CD86) [43, 44]. Third, IL-2 is absorbed rapidly by the activated PD-1^+^ Treg cells and is kept away from effector T cells in the tumor response [41]. Fourth, upon ICI treatment, many cytokines are upregulated to promote Treg cell expansion and induction, such as adenosine and IDO (indoleamine 2,3-dioxygenase) [40]. Fifth, IFN-γ is suppressed by Treg cells through inhibition of the cytotoxic activities of immune effector cells (Fig. 3). In general, the antitumor efficacy of ICI may be regulated by PD-1^+^ Treg cells and effector T cells. In HPD, tumor cells would grow uncontrollably as a result of the dominant status of PD-1^+^ Treg cells and escape of effector T cells from killing.

##### Exhausted T cells

T cell dysfunction is defined as T cell exhaustion, with inhibitory receptors strongly upregulated in diversity and number, such as PD-1, LAG3 (lymphocyte activation gene 3 protein), TIGIT (T cell immunoreceptor with immunoglobulin and ITIM domains) and TIM3 (T cell immunoglobulin and mucin domain-containing protein 3). Additionally, the dysfunction of T-cells includes diminished cytokine production, an impaired kill-effect and hypoproliferation.

**Fig. 4:**
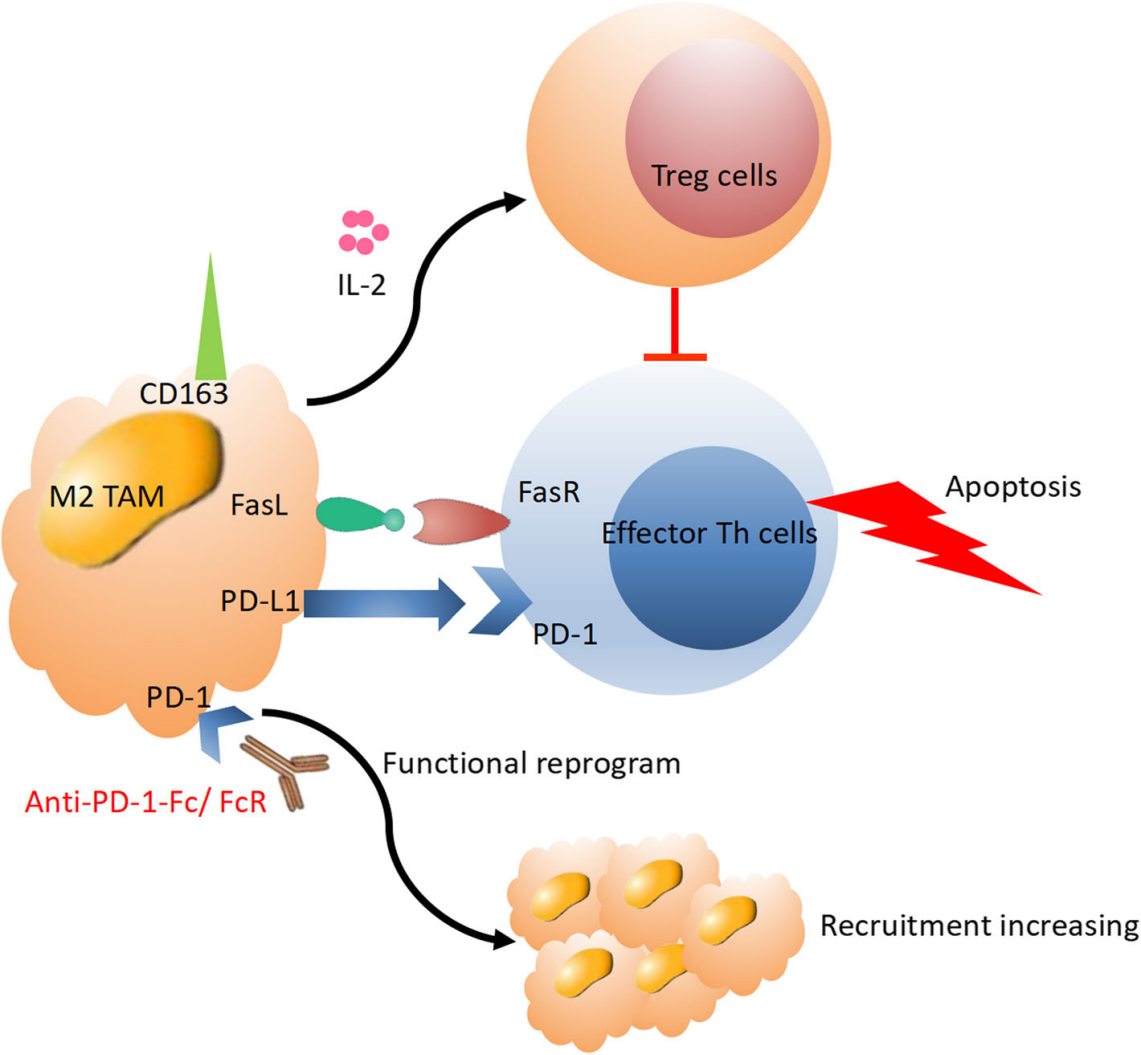
T-cell exhaustion in patients with HPD. On the basis of a high concentration of neoantigen, the upregulation of exhausted T cells with high coexpression of PD-1, LAG3, TIM3, CTLA4 and TIGIT is connected to the resistance to anti-PD-1 therapy. Furthermore, exhausted T cells fail to reinvigorate as memory T cells ultimately become re-exhausted upon antigen clearance

Upon ICI therapy, the compensatory immune-checkpoints might overexpress and regulate local immune suppression and escape [45]. When tumor progression occurred with the use of ICI in two NSCLC patients and a lung adenocarcinoma murine model, TIM3 overexpression was found in tumor-infiltrating CD8^+^ T cells. Similarly, CTLA-4 and LAG3 on cytotoxic CD8^+^ T cells were upregulated as a result of PD-1 blockage in a murine model of ovarian cancer [46]. With expression of the above compensatory receptors, CD8^+^ T cells show serious dysfunction in cytokine production, proliferation and migration. Furthermore, due to the high concentration of neoantigens, the exhausted T cells of patients with HPD were eventually re-exhausted and failed to reinvigorate as memory T cells that work on the clearance of old antigens. In addition, T cells could be inhibited by the combination of PD-1/PD-L2 during ICI therapy, while PD-1 or CD80 usually does not bind with PD-L2 (expressed on endothelial cells, macrophages and DCs) on the surface of T cells [47] (Fig. 4).

##### Dendritic cells

APCs (antigen-presenting cells), especially DCs (dendritic cells) play a crucial role in generating the anti-tumor response by T cells through indirect uptake of tumor antigen, which occurs in the tumor site or the lymph nodes tracked by costimulation and activation of antigen-specific T cells. In particular, the interaction between PD-1 on T cells and PD-L1 on DCs could bring about inhibition of anti-tumor responses along with the presentation of tumor antigens [48]. Additionally, the cytokines in the tumor microenvironment have a potential interference effect on DCs. The influence of TGF-β on DCs impairs their migratory ability and decreases the expression of costimulatory molecules (CD86/80) [49, 50]. IL-6 has an effect on preventing DC maturation through the IL-6-gp130-STAT3 axis [51]. IDO plays a role in inhibiting the activity of DCs. Thus, the alteration of DCs has a negative regulatory effect on T cells in the anti-tumor response [52]. In HPD, the role of DCs still needs to be validated.

##### Myeloid-derived suppressor cells

MDSCs (myeloid-derived suppressor cells) represent a heterogeneous group of immunosuppressive cells, which have been thought to play a vital role in regulating immune responses in many pathological conditions, such as tumors and chronic infection. Especially in the progression of tumors, MDSCs contribute to tumor angiogenesis, metastasis and prognosis [53]. Various findings have shown that low levels of MDSCs are associated with better OS in prostate cancer [54] and breast and colorectal cancer [55]. Interestingly, MDSCs have been found to be related to the response to ICI. Meyer et al. [56] reported that melanoma patients with low frequencies of MDSCs might benefit from ipilimumab treatment, which could be used as a predictor in ICI treatment. Similar conclusions have been reported in other studies [57–59]. Furthermore, in mouse models of renal cell and Lewis lung carcinoma, the combination of MDSCs blockade and ICI therapy was found to result in a better OS than ICI therapy alone [60]. Meanwhile, the combination with inhibitors, including anti-CFS-1R, gemcitabine, phenformin, and entinostat, enhances the effect of ICI therapy in melanoma [61] and head and neck cancer [62] (resistance to CTLA-4 checkpoint inhibition reversed through selective elimination of granulocytic myeloid cells). However, the mechanism of MDSCs in HPD is still unknown but might be related to impairment of the activity of effector T cells, inducing expansion of Treg cells, reducing the functions of NK cells, and secretion of cytokines (such as IDO, VEGF and MMP9) [63].

##### TAM (M2) cells

M2 tumor-associated macrophages (TAMs) are a subset of TAMs that participate in the response to immunotherapy. They are associated with pro-tumorigenic properties, while M1 macrophages promote anti-tumor immunity. Clinical studies found that the presence of M2 macrophages is correlated with a poor prognosis in various tumors [64]. Tumor growth might be inhibited by depletion of TAMs in a murine model of lung adenocarcinoma, especially by a decrease in M2 TAM recruitment due to disruption of CCR2 activation and CCL2 signaling [65].

**Fig. 5:**
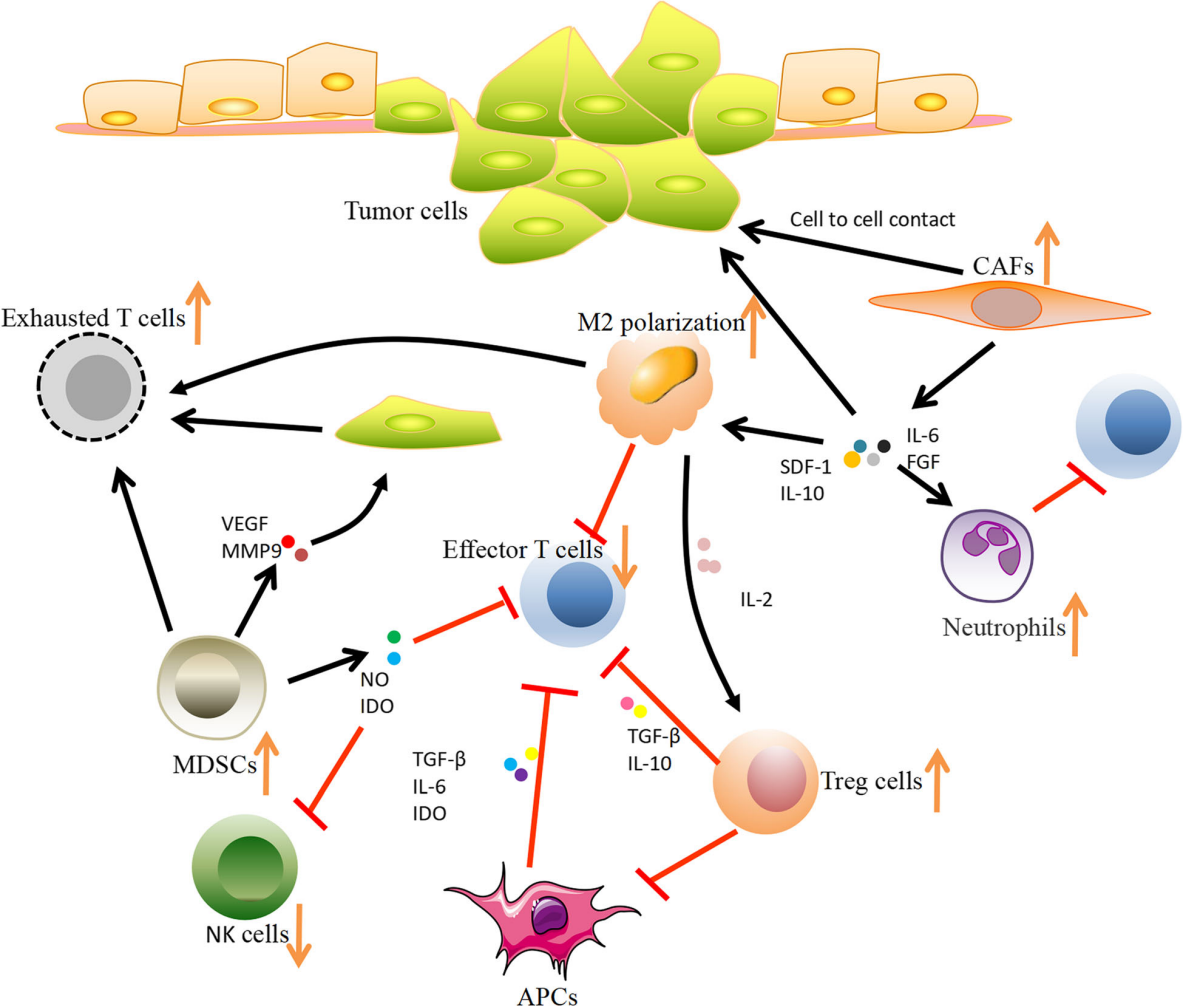
In the HPD tumor microenvironment, immunosuppression activity might be stimulated by interaction with M2 TAMs upon PD-1/PD-L1 blockade treatment through activation of Treg cells, promoting apoptosis of effector T cells and increasing recruitment of M2 TAMs

A study of hepatocellular carcinoma suggested that the responses of T cells were directly suppressed by macrophages. Blocking the binding of macrophage colony-stimulating growth factor with receptors such as CSF-1R could lead to tumor rejection. Increasing IFN production and decreasing M2 TAM frequencies helped in overcoming the potential resistance of TAMs in a murine model of pancreatic cancer. Furthermore, tumor regression could be strengthened by blocking CSF-1R with a combination of PD-1 and/or CTLA-4 antibodies [66]. Lo Russo G et al. reported that infiltration of M2 TAMs (M2-like CD163^+^CD33^+^PD-L1^+^ clustered epithelioid macrophages) was increased after treatment with PD-1 antibody, along with a large increase in tumor progression [11]. They assumed that the binding between FcR and the specific immunophenotype of ICI might trigger clustering of M2 TAMs and result in functional reprogramming of M2 TAMs toward a more invasive protumorigenic phenotype. Thus, HPD tends to be induced in patients with particular genetic and immune profiles. Alternative immune pathways in HPD need to be elucidated in further research (Fig. 5).

#### Nonimmunological cells

##### CAFs

Cancer associated fibroblasts (CAFs) are an important member of the tumor microenvironment and have the specific marker alpha smooth muscle actin (α-SMA). As a subset of activated fibroblasts, CAFs play a positive role in tumor growth, progression, metastasis and drug resistance, similar to myofibroblasts [67–69]. Many studies have reported that CAFs have an influence on regulation of immune cell recruitment, cell-cell interactions with tumor cells, secretion of a variety of cytokines and remodeling of the tumor matrix. Gok et al. [70] reported that CAFs are related to immunosuppression through recruitment of monocytes and transdifferentiating M2 TAMs in breast cancer cells and thus could be used as an immune therapeutic target in the future. The main explanation in ICB is that CAFs could induce PD-1 expression in immune cells. Other explanations include the following: monocytes are recruited by CAFs, which markedly encompass immunosuppression and enhance the motility of tumor cells; CAFs are capable of inducing differentiation of M1 TAMs into M2 TAMs and thus are related to the number of M2 TAMs. Cheng et al. [71] found that the function of T cells was impaired by immunosuppressive neutrophils, which were induced by CAFs in HCC. They suggested that CAFs recruit neutrophils that then released IL-6, which could induce neutrophil activation and PD-L1 expression; thus, the PD-L1^+^ activated neutrophils have a pro-tumor role through the IL6-STAT3-PD-L1 signaling pathway to inhibit T cell immunity. Other notions have been presented about the role of CAFs through TGF-β signaling, such as promoting the differentiation of Treg cells, and inhibiting the activation of CTLs and NK cells. Furthermore, CAFs might induce PD-L1 expression in tumor cells. In ICI treatment, CAFs may be a novel point for immunosuppression related to HPD.

#### *C*ytokines and inflammatory factors: the interferon-γ (IFN-γ) pathway

The relationship between anti-PD-1/PD-L1 treatment and the immune microenvironment is regulated by the expression of IFN-γ [72]. The immune-resistance of tumors could be enhanced by IFN-γ through promotion of PD-L1 expression in cancer cells. Activation of JAK1/JAK2 leads to the recruitment and phosphorylation of STAT1/STAT3, followed by the binding of IFN-γ to its specific receptor IFNGR1/IFNGR2. Then, expression of PD-L1 could be enhanced by activation of interferon regulatory factor 1 (IRF1) [73]. Moreover, through a JAK2-independent pathway, the PI3K-AKT pathway could be activated by IFN-γ, thus upregulating PD-L1 expression in lung adenocarcinoma [74]. Tumor cells could escape from the immune response by increasing the PD-L1 level, and thus, the immune-resistance of tumors could be inhibited by ICI treatment. Meanwhile, the improvement in antigen-presenting machinery (APM) components and chemokine production induced by IFN-γ attracted T cells and had a suppressive effect on tumor growth. Sustained release of IFN-γ by T cells could upregulate the selection pressure of tumor cells, resulting in acquired deficiency of the IFN-γ pathway and acquired resistance to ICI through loss of sensitivity to IFN-γ.

##### Activation of compensatory immune checkpoints in T cells

In vitro studies show that preneutralization of PD-1 antibody and soluble PD-1 might be prevented through an increase in IL-10 secretion and inhibition of the activation of bone marrow-derived DCs by soluble PD-1-Ig fusion protein [75]. In conclusion, the immune response of antitumor CD8^+^ T cells against cancer cells might be inhibited by upregulation of other alternative checkpoints in an immunosuppression mechanism.

#### Abnormality of laboratory biomarkers

As a noninvasive and easy-to-use method, serum biomarker detection has been used to screen various tumors. Only a few studies have determined whether serum features could be used to monitor HPD. Sasaki et al. [76] found that the ANC (absolute neutrophil count), CRP (C-reactive protein) and LDH (lactate dehydrogenase) levels were obviously lower (ANC, 2720/μl vs. 4490/μl, *P* = 0.002; CRP, 0.50 mg/dl vs. 4.0 mg/dl, *P* = 0.006; LDH, 179.5 U/l vs. 396.0 U/l, *P* = 0.006) in non-HPD patients than in HPD patients among 73 patients administered nivolumab treatment. Especially, the levels of ANC (7740/μl vs. 4490/μl) and CRP (8.3 mg/dl vs. 4.0 mg/dl) were significantly increased in the HPD group after 4 weeks of nivolumab treatment. Upregulation of the ANC might be used to reflect the release of premature myeloid cells from the bone marrow, which modulates crucial cancer-associated activities, such as immune evasion and cancer therapy options. Most importantly, the response to ICI treatment is related to MDSCs recruitment [77]. Gonda et al. [78] reported that MDSCs counts are also positively related to CRP levels. Thus, Sasaki A proposed that elevated ANC and CRP levels might be caused by an increase in MDSCs fractions, which could potentially be used to assess HPD status [76]. Although the relevant mechanisms are poorly understood, detection of alterations in these laboratory biomarkers can be a favorable and easy approach for early HPD prediction.

### Clinical indicators of HPD

To date, certain clinical variables have been found to be associated with HPD, including local recurrence in the field of irradiation, two or more metastatic areas and advanced age.

Saada-Bouzid et al. [15] reported for the first time that HPD occurred upon ICI treatment in RSCCHN patients with local recurrence, while HPD did not occur in patients with distant recurrence or without local recurrence. An important confounding hypothesis of HPD is that early metastasis in the neck lymph nodes may reinforce the immune reaction. Thus, it seems reasonable to postulate that the tumor microenvironment is modified by radiotherapy by downregulating the main cytokines, decreasing TIL (tumor infiltrating lymphocyte) numbers and increasing the number of PD-L1 transcripts [79]. Furthermore, based on a T-cell dependent pathway, the failure of ICI treatment is thought to be significantly related to a lack of TILs [80]. However, some retrospective models have shown that the immune system could be stimulated or inhibited by various doses and delivery methods [81].

Later, Ferrara et al. [13] suggested that HPD occurred more frequently in advanced NSCLC patients with more than two metastatic sites during therapy with PD-1/PD-L1 antibody. One possible explanation is that more aggressive tumor phenotypes imply a higher risk of HPD. However, there are inconsistent studies indicating that the progression rate and new lesion incidence were lower at the beginning of ICI treatment, and thus, more efforts should be made to explore the possible relationship between metastatic sites and HPD occurrence.

Third, older patients (≥65) were found to undergo HPD during ICI treatment more often than younger patients and had a worse prognosis [12]. Many randomized controlled trials also reported that less benefit was obtained by older patients than by younger patients, which validated that age plays a role in immunotherapy [82]. The mechanisms connecting HPD and advanced age remain unclear. Immunosenescence is one possibility. In older individuals, as one of the most obvious characteristics, T cell immunity reduction generated by age-related thymic atrophy has been thought to be connected with autoimmune disease, inflammation and tumorigenesis. Those diseases could be caused by decreasing antigen recognition and a weak immune system. For example, decreases in native T cells along with age are produced by the thymus throughout life [83]. Furthermore, while the number of T cells is maintained at a certain level, the diversity of T cells is downregulated with age: memory T cells are consistently upregulated with age, while effector T cells are obviously downregulated, with a specific decrease in the diversity of T cell receptors and greatly increased potential of decreased proliferation [84]. Signaling of T cells, such as by the T cell receptor also wanes in aged patients [85]. As a potential predictive biomarker, the role of age in HPD needs further study in a great number of patients.

### Remaining HPD controversies

Given that HPD has been reported in several studies after anti-PD-1/PD-L1 therapies, we wonder if this phenomenon is a clinical reality in response to immunotherapy for cancer or a simple behavior of the natural outcome of aggressive, fast-growing tumors without treatment? This question, was also proposed in the 2019 Annual Meeting of AACR American (Association for Cancer Research) in Atlanta, GA, and different views exist: HPD was supported by 58% of participants, 12% of participants held other views, and the other 30% were undecided.

One reason for skepticism about HPD is the lacking of an accurate definition, although the key criteria for HPD are fast progression and early death. For fast progression, TGR/TGK are used to measure tumor burden on CT scans before and after ICI. However, there are still several limitations: the cut-off for TGR is not unified, a lack of CT-scan evaluations in the first line of treatment, the growth rate of the tumor is not included in the TGR estimation, and the risk of HPD with/without overlapping other therapies is increased; thus, TGK should be included. The definition of HPD is a more than two-fold increase in TGR and TGK between the basic evaluation and the first CT imaging evaluation in the period of anti-PD1/PD-L1 therapy based on RECIST 1.1 of PD, which can effectively eliminate the possibility of overestimating the incidence of HPD. Based on TGR and TGK, the norm of tumor growth only includes the variation in target lesions, but new lesions are excluded. Because TGR and TGK are very similar (measured by CT) in a robust manner, TTF has also been employed as one of the clinical criteria. In the 2019 AACR, the criteria were broadly agreed to be a TTF within 2 months, more than a two-fold tumor burden compared with the baseline TGK, and at least a two-fold increase in the pace of disease progression. Essentially, there must be an inflection point in the slope of TGK.

In addition, as far as we know, previous studies of HPD have been retrospective and only analyzed a small number of patients with diverse cancer types. All these studies reported that the incidence rates of HPD have no statistical variation among cancer types. Prospective studies in various tumors are needed to validate these findings.

Furthermore, some researchers doubt that all the treatment strategies may affect the pattern of therapeutic response. As we know, HPD patients have a significantly shorter OS compared with other patients [13], suggesting that HPD is an unexpected therapeutic event. There is no significant difference in the OS of HPD patients with ICI therapy and chemotherapy (median (95% CI): 3.4 (2.8–7.5) VS. 4.5 (2.5–6.5) months, *P* > 0.05) [13]. In addition, there is no specific data on the occurrence of HPD after traditional chemotherapy, and few data are available on HPD in patients treated with combination therapies or not treated.

Fourth, biological mechanisms and predictive factors are poorly understood. To better prevent HPD, multidimensional studies should be performed, such as gene-expression patterns studies (whole-genome sequencing in tumor cells), cellular pathology studies (expression of immune-checkpoints and/or their ligands), radiometric studies (high-throughput CT, MRI, and PET scans), and studies of serum and tumor microenvironment biomarkers (quantification of different cellular populations, cytokines and soluble mediators).

### Management of HPD

Effective strategies should be explored to treat patients with HPD. Patients showing HPD clinical features should suspend further ICI treatment, and those in good clinical condition should be re-estimated early and switched to another potentially effective treatment, such as chemotherapy. However, the rapid deterioration in HPD limits the opportunity to administer other treatments to patients. Due to the lack of relevant literature reports, whether chemotherapy might have an influence on the OS of patients with HPD remains unknown. Previous retrospective studies have only reported that the response to chemotherapy following progression under immunotherapy was not ideal [86, 87]. Based on promotion of tumor progression, cytotoxic and/or anti-angiogenic agents might also be connected with anti-tumor efficacy. Many studies on the tumor immune microenvironment will enable the identification of specific subsets of immunosuppressive cells and alternative immune checkpoints for further exploration of immunomodulatory agents in the near future. Moreover, researchers should attempt to assess the HPD status at the time of ICI usage and guide the best management of patients. Earlier identification of HPD will allow patients to benefit more from alternative therapies.

## Conclusion

In the past few years, the advantage of ICI therapy in oncology has led to a prominent improvement in the prognosis of patients with several tumor types. However, a growing body of data suggests that some patients (9–23% patients) experience an inconceivable acceleration in tumor growth during anti-PD-1/PD-L1 therapy. This view that a given antitumor therapy might stimulate tumor progression is not new but is still controversial. The definition of HPD should be standardized in the future. In addition to the currently used criteria, further methods to assess the response to ICI therapy need to be proposed to identify and guide reasonable management of patients with HPD. Multidimensional studies should be conducted to integrate an optimal method to assess HPD at an earlier time point than currently in use, thereby avoiding the risk of patients receiving ineffective treatment. Further investigations in larger cohorts and prospective studies are needed. In addition, there is an urgent need to explore the molecular mechanism of HPD, which will lead to effective biomarkers for identifying people at high risk for HPD.

## Abbreviations

ICI: Immune checkpoint inhibitors
NSCLC: Non-small cell lung cancer
HNSCC: Squamous cell carcinoma of the head and neck
PR: Partial response
CR: Complete response
OS: Overall survival
HPD: Hyperprogressive disease
TTF: Time to treatment failure
TGR: Tumor growth rate
TGK: Tumor growth kinetics
PFS: Progression-free survival
MDM2/4: Murine double minute 2/4
MSI: Microsatellite instability
IRF-8: Interferon regulatory factor 8
MMR: Mismatch repair
TMB: Tumor mutational burden
APCs: Antigen-presenting cells
DCs: Dendritic cells
MDSCs: Myeloid-derived suppressor cells
TAMs: Tumor-associated macrophages
CAFs: Cancer-associated fibroblasts
ANC: Absolute neutrophil count
CRP: C-reactive protein
TILs: Tumor-infiltrating lymphocytes
